# Inter-Organelle Membrane Contact Sites and Mitochondrial Quality Control during Aging: A Geroscience View

**DOI:** 10.3390/cells9030598

**Published:** 2020-03-03

**Authors:** Anna Picca, Riccardo Calvani, Hélio José Coelho-Junior, Francesco Landi, Roberto Bernabei, Emanuele Marzetti

**Affiliations:** 1 Fondazione Policlinico Universitario “Agostino Gemelli” IRCCS, 00168 Rome, Italy; anna.picca@guest.policlinicogemelli.it (A.P.); francesco.landi@unicatt.it (F.L.); emanuele.marzetti@policlinicogemelli.it (E.M.); 2 Institute of Internal Medicine and Geriatrics, Università Cattolica del Sacro Cuore, 00168 Rome, Italy; coelhojunior@hotmail.com.br

**Keywords:** biomarkers, exosomes, extracellular vesicles, geroprotective interventions, mitophagy, mitochondrial damage, mitochondrial dynamics, mitochondrial-derived vesicles, mitochondrial-lysosomal axis, neurodegeneration

## Abstract

Mitochondrial dysfunction and failing mitochondrial quality control (MQC) are major determinants of aging. Far from being standalone organelles, mitochondria are intricately related with cellular other compartments, including lysosomes. The intimate relationship between mitochondria and lysosomes is reflected by the fact that lysosomal degradation of dysfunctional mitochondria is the final step of mitophagy. Inter-organelle membrane contact sites also allow bidirectional communication between mitochondria and lysosomes as part of nondegradative pathways. This interaction establishes a functional unit that regulates metabolic signaling, mitochondrial dynamics, and, hence, MQC. Contacts of mitochondria with the endoplasmic reticulum (ER) have also been described. ER-mitochondrial interactions are relevant to Ca^2+^ homeostasis, transfer of phospholipid precursors to mitochondria, and integration of apoptotic signaling. Many proteins involved in mitochondrial contact sites with other organelles also participate to degradative MQC pathways. Hence, a comprehensive assessment of mitochondrial dysfunction during aging requires a thorough evaluation of degradative and nondegradative inter-organelle pathways. Here, we present a geroscience overview on (1) degradative MQC pathways, (2) nondegradative processes involving inter-organelle tethering, (3) age-related changes in inter-organelle degradative and nondegradative pathways, and (4) relevance of MQC failure to inflammaging and age-related conditions, with a focus on Parkinson’s disease as a prototypical geroscience condition.

## 1. Introduction

The comprehension of biological pathways involved in organismal aging and age-related conditions has been a chimera for biogerontologists [1]. The several hypes and falls that have characterized this field of research deep their roots into the complex nature of aging itself. Indeed, during aging, multiple inter-related processes co-occur, producing a multitude of different and stochastically determined phenotypes [2]. Though, some conserved phenomena have been identified as major biological drivers of aging, the so-called “hallmarks of aging” [3]. These include mitochondrial dysfunction, loss of proteostasis, cellular senescence, altered intercellular communication, genomic instability, telomere attrition, epigenetic alterations, deregulated nutrient sensing, and stem cell exhaustion [3]. According to the geroscience hypothesis, perturbations in these mechanisms increase the susceptibility to most chronic diseases, functional loss, and eventually, death [4]. Hence, these biologic pillars represent ideal targets for interventions to foster healthy aging [5,6].

Mitochondrial dysfunction has attracted considerable interest as a target for geroprotective interventions. Indeed, mitochondria play sensor-transducer-effector roles in a multitude of biological processes, including integration of cell death signaling and preservation of cell stemness [7,8]. Albeit long considered to be standalone organelles, a great deal of evidence indicates that mitochondria interact physically and functionally with other cellular compartments via membrane contact sites and tethering molecules [9,10]. In particular, mitochondria establish connections with the endosomal compartment [11,12] and lysosomes [13,14]. These interactions support cytosolic shuttle systems of ions and metabolites across organelles [10,15], and participate to the regulation of cellular housekeeping processes [13,14].

The mitochondrial-lysosomal axis is a major actor in mitochondrial quality control (MQC), a hierarchical network of pathways that ensure organellar homeostasis through the coordination of mitochondrial proteostasis, dynamics, biogenesis, and autophagy [16]. While continuous cycles of fusion and fission preserve mitochondrial shape and dilute damage along the network [17] mitochondrial hyper-fission segregates damaged or unnecessary organelles from the network [17]. Severely damaged mitochondria are subsequently disposed via a selective form of autophagy referred to as mitophagy [18]. Cleared mitochondria are eventually replenished via biogenesis to maintain an adequate mitochondrial pool within the cell [19]. Damaged mitochondrial components may follow an alternative degradative route that operates through the release of specialized extracellular vesicles (EVs), namely mitochondrial derived vesicles (MDVs), before whole-sale organelle degradation is triggered. This pathway involving mitochondrial-lysosomal crosstalk has been proposed as an additional layer of MQC [20].

Dysregulation of mitophagy and disruption of the mitochondrial-lysosomal axis coupled with abnormal EV secretion have been implicated as mechanisms in the aging process and related disease conditions [16,21]. More specifically, the garbage theory of aging poses that damaged mitochondria, protein aggregates, and lipofuscin accumulate as a result of inefficient cellular quality control [22]. The progressive accrual of intracellular “waste” further depresses cell recycling processes, thereby impinging on cell homeostasis and tissue integrity [22].

A role for inter-organelle membrane contact sites of mitochondria with lysosomes and lysosome-related organelles distinct not pertaining to MQC has recently been described [23]. These interactions occur as part of nondegradative pathways to support transfer of lipids, Ca^2+^, and iron between organelles, and to regulate mitochondrial fission [24].

Here, we discuss (1) degradative pathways involved in MQC with a special focus on mitophagy and pathways entailing EV generation, (2) nondegradative processes involving inter-organelle contact sites, (3) age-related changes in inter-organelle degradative and nondegradative pathways and their possible exploitation for therapeutic purposes, and (4) the relevance of MQC failure to inflammaging and neurodegeneration, with a focus on Parkinson’s disease (PD) as a prototypical geroscience condition.

## 2. Mitochondrial-Lysosomal Membrane Contact Sites

### 2.1. Degradative Pathways

Fine-tuning of MQC processes is key to preserving a functional mitochondrial network within the cell [16]. Mitochondrial fission regulates the rate of mitochondrial biogenesis and mitochondrial DNA (mtDNA) synthesis [25,26] under the control of GTPase dynamin-related protein 1 (DRP1), fission protein 1 (FIS1), dynamin 2, and actin [24,27,28,29]. Mitochondrial membrane tethering and fusion, which are mediated by the outer membrane GTPases mitofusin (MFN) 1 and MFN2 and by the inner membrane GTPase optic atrophy 1 (OPA1), enable mixing of mitochondrial proteins, mtDNA, and metabolites and allows diluting mitochondrial damage along the network [25].

Functional connections between lysosomes and mitochondria have also been described [15]. Indeed, defects in either of the two organelles induce impairments in the other, indicating the existence of a mitochondrial-lysosomal axis [30]. The genetic ablation of mitochondrial transcription factor A (TFAM), responsible for mtDNA replication, transcription and maintenance [19], increases the number of lysosomes in T cells [30]. However, lysosomal activity is impaired when deficient mitochondrial respiration and disruption of endolysosomal trafficking occur, suggesting a link between primary mitochondrial dysfunction and lysosomal storage disorders [30]. On a similar note, ablation or pharmacological inhibition of apoptosis inducing factor (AIF), OPA1, or phosphatase and tensin homolog (PTEN)-induced putative kinase 1 (PINK1) in neurons impairs lysosome activity, thereby causing accrual of autophagic substrates [31]. Moreover, the restoration of lysosomal pH by lysosome-targeted nanoparticles reinstates mitophagy in pancreatic cells exposed to high concentrations of free fatty acids [32]. These findings indicate that, at least under lipotoxic conditions, mitochondrial dysfunction develops downstream of lysosomal alkalization and that recovery of lysosomal acidity restores MQC [32].

#### 2.1.1. Mitophagy

The selective removal of whole mitochondria through mitophagy involves a multistep process that begins with the engulfment of damaged mitochondria by an autophagosome. The latter fuses with lysosomes to form an autolysosome where the content is degraded [18]. This sequence of events is orchestrated by a sophisticated molecular machinery [33]. While the removal of dysfunctional mitochondria via mitophagy has been described in different mammalian cells, the output of the process differs depending on the cell. Complete mitophagy is, indeed, observed during erythrocyte maturation, while selective degradation of sperm-derived mitochondria occurs after oocyte fertilization [34,35,36]. Regardless of cell specificity, the tagging of damaged mitochondria to mitophagy via ubiquination is required for their subsequent interaction with mitophagy receptors such as nuclear dot protein 52 (NDP52) and optineurin (OPTN) [37,38]. The preparation of dysfunctional organelles for disposal is coordinated by the mitochondrial protein kinase PINK1 and the ubiquitin E3 ligase, Parkin [39,40]. Following mitochondrial depolarization, the activation of PINK1 guides the recruitment of Parkin, a cytosolic protein, on depolarized mitochondria [41,42,43,44]. This process is enabled by PINK1-mediated phosphorylation and ubiquitination of Parkin at serine 65 [45,46]. These modifications ensure maximal recruitment and activation of Parkin at the sites of damaged mitochondria [47,48,49]. Once recruited, Parkin itself ubiquitinates several proteins located at the mitochondrial outer membrane interface in order to mediate the subsequent sequestration of mitochondria into the isolation membrane via interaction with specific adaptor proteins [50]. The accrual of the ubiquitin-binding adaptor protein p62/sequestosome-1 on depolarized mitochondria and the subsequent binding to the microtubule-associated protein 1A/1B-light chain 3 (LC3) facilitate the delivery of damaged mitochondria to autophagosomes to complete the degradative process [50].

In addition to PINK and Parkin, the Ras-associated binding protein 7 (RAB7), a protein belonging to the Ras-like GTPase superfamily, is a relevant player in mitophagy [51]. The main functions of this small GTPase are to (1) control maturation of early endosomes, (2) regulate transport of intracellular material from late endosomes to lysosomes, (3) supervise lysosomal biogenesis, and (4) enable clustering and fusion of late endosomes and lysosomes in the perinuclear region of the cell [52]. RAB7 swings between an active, lysosomal-localized GTP-binding state and an inactive, cytosolic GDP-binding state. Via its alternate active and inactive state, RAB7 modulates the tethering and untethering of mitochondrial-lysosomal contact sites. In particular, a contact site between the two organelles can be established via lysosomal GTP-bound RAB7 that may be tethered to mitochondria via its binding to a RAB7 effector protein. Notably, the expression of a constitutively active GTP-bound form of RAB7 that is unable to undergo GTP hydrolysis (RAB7 Q67L) increases the number of mitochondrial-lysosomal contacts and prolongs the time of membrane tethering [14].

Together with the Tre-2/Bub2/Cdc16 (TBC) domain family, member 15 and 17 (TBC1D15/TBC1D17) and FIS1, RAB7 is an effector of mitophagy downstream of Parkin and is involved in autophagosome biogenesis during mitophagy [51]. TBC1D15 and 17 belong to the family of TBC proteins containing RAB-specific GTPase-activating protein (RABGAP) functions [53,54]. FIS1, instead, is a protein anchored to the mitochondrial outer membrane by its C-terminal domain deputed to assist in mitochondrial fission [55]. When TBC1D15 is depleted or its RABGAP activity is lacking, accumulation of LC3-tagged phagosomes without cargo orientation occurs. As a consequence, an elongated structure departs from mitochondria along microtubule tracks [51]. Therefore, the interaction of TBC1D15/17 with LC3 and FIS1 is crucial for coordinating RAB7 activity and guiding the preautophagosomal isolation membrane that selectively engulfs damaged mitochondria [51]. Moreover, silencing of RAB7 suppresses the abnormal LC3 accumulation and tubulation in TBC1D15 cells [51].

Taken as a whole, these findings indicate that, while constitutive RAB7 activity favors the expansion of the LC3-positive isolation membrane, RAB7 inactivation may be required for the release of LC3-bound membranes from microtubules [51,56]. This attributes RAB7 an additional role besides its function of controlling the final step of maturation of autophagosomes by their fusion with lysosomes [57,58]. The interaction between the mitochondrial fusion-related protein MFN2 and RAB7 increases in response to starvation, which may suggest the involvement of RAB7 as an adaptor protein used by MNF2 during maturation of the autophagosomal membrane [59]. Thus, RAB7 seems to support both autophagosome formation and maturation during mitophagy.

The insufficient clearance of damaged mitochondria through mitophagy is acknowledged as a major mechanism driving cell senescence and organismal aging [60]. The persistence of dysfunctional mitochondria is especially detrimental to long-lived cells (e.g., neurons, cardiac and skeletal myocytes, and T lymphocytes) that cannot efficiently dilute organellar damage through cell division. This phenomenon is invoked as a possible explanation to the fact that brain, heart, skeletal muscle, and immune system are particularly vulnerable to dysfunction during aging [60]. Indeed, giant, irregularly shaped mitochondria are frequently encountered in aged post-mitotic cells, where they can displace normal mitochondria, ultimately leading to extensive oxidative stress and energy failure [61,62]. A primary defect in fission has been proposed as a mechanism underlying the formation of giant mitochondria that would be less likely to be autophagocytosed because of their bulk dimensions [61]. Alternatively, hyperfused mitochondria with reduced respiration rate might suffer milder oxidative damage on their own membranes and, consequently, be less targeted to mitophagy that well-functioning mitochondria [63]. This latter view has recently been refined following the discovery of an alternative route for disposing mildly damaged mitochondria through MDV generation and release [64,65]. As discussed in the following section, MDVs may serve as a first line of defense through which mitochondria extrude damaged components to avoid organellar failure.

#### 2.1.2. Generation and Release of Mitochondrial Derived Vesicles

The generation and release of MDVs, small vesicles of ~100 nm in diameter [66], have been proposed as an additional pathway to allow degradation of organellar components via delivery to lysosomes [67]. MDVs are generated through the selective incorporation of protein cargoes, including outer and inner membrane proteins and matrix content [65,67,68]. The molecular determinants of MDV generation are still unclear. However, MDV biogenesis seems to proceed independent of DRP1 and to require priming by PINK1 and Parkin [66]. A working hypothesis on MDV formation implies that, under oxidative stress conditions, the accumulation of protein aggregates in proximity to mitochondrial membranes concomitant with cardiolipin oxidation generates unusual membrane curvatures [66]. This would interfere with the function of mitochondrial import channels, followed by accumulation of PINK1 that ubiquinates and recruits Parkin. Eventually, a vesicle is formed and released through a process relying on yet unidentified proteins [66]. MDVs can face two distinct fates depending on whether they are targeted to the late endosome/multivesicular body for degradation [67] or to a subpopulation of peroxisomes possibly for cargo detoxification [68]. Accordingly, MDV generation may pertain to degradative pathways. Down this road, large double-membrane vesicles enriched in mitochondrial components are released. Hence, PINK1 and Parkin represent a point of convergence for MDV generation and mitophagy. Different from mitophagy, this shuttle system does not require mitochondrial depolarization, autophagy signaling, or mitochondrial fission [67]. Indeed, MDVs are generated also in cells lacking autophagy-related serine/threonine kinase gene (Atg) 5, Beclin-1, or RAB9, as well as after DRP1-silencing [67]. Thus, MDV generation and delivery are thought to complement mitophagy for MQC when mitochondrial damage is mild or when mitophagy is overwhelmed or compromised [69]. The presence of mitochondrial constituents within exosomes is itself an indirect evidence of crosstalk between mitochondria and the endolysosomal system [70,71].

**Figure 1:**
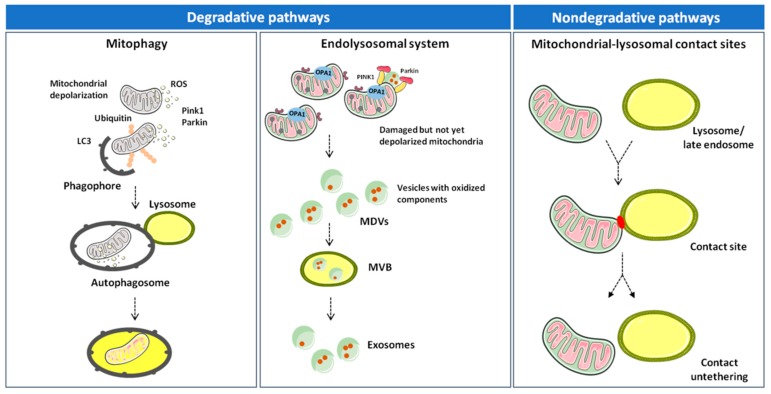
Degradative and nondegradative pathways in mitochondrial quality control. Mitochondrial dynamics are ensured by several factors that regulate fusion (mitofusin (MFN) 1, MFN2, and optic atrophy 1 (OPA1)) and fission (dynamin-related protein 1 (DRP1) and mitochondrial fission 1 protein (FIS1)). While fusion dilutes organellar damage along the network, fission targets dysfunctional mitochondria and triggers their clearance through mitophagy in a phosphatase and tensin homolog (PTEN)-induced putative kinase 1 (PINK1)/Parkin–dependent manner. Mildly damaged mitochondria and organellar components may be recycled via the generation of mitochondrial derived vesicles (MDVs). Once formed, MDVs reach out the endolysosomal system, form multivesicular bodies (MVBs), and are released into the extracellular space as exosomes. Abbreviations: LC3, microtubule-associated proteins 1A/1B light chain 3 and ROS, reactive oxygen species.

Altogether, the available evidence suggests that inter-organelle mitochondrial-lysosomal membrane contact sites enable a fine coordination between mitophagy and MDV-mediated degradative pathways. In this context, mitophagy disposes dysfunctional mitochondria as an extreme attempt to maintain cell homeostasis [18,72]. MDV generation may serve as an alternative route to clear nonirreversibly damaged organelles and dispose mitochondrial components before whole-sale organelle degradation is triggered (Figure 1) [64,65].

At the systemic level, the release of damaged mitochondrial components within MDVs may contribute to sterile inflammation, an inflammatory response mounted in the absence of infections [73]. This process is organized within the framework of innate immune response and has been included as part of the ‘‘danger theory’’ of inflammation [74]. According to this view, misplaced noxious material from injured cells (i.e., damage-associated molecular patterns (DAMPs)) triggers caspase-1 activation and the secretion of pro-inflammatory cytokines [75]. The release of MDV content (e.g., mitochondrial proteins, mtDNA) can elicit several inflammatory pathways by interacting with (1) Toll-like receptors (TLRs), (2) Nod-like receptor (NLR) family pyrin domain containing 3 (NLRP3) inflammasome, and (3) cytosolic cyclic GMP-AMP synthase (cGAS)-stimulator of interferon genes (STING) DNA sensing system [76].

During aging, the persistence of sterile, low-grade inflammation—a condition called “inflammaging”—is believed to contribute to the progression of the aging process and to the pathogenesis of age-related diseases [77,78]. Relevant pathways elicited upon MDV release during aging are discussed in Section 4.

### 2.2. Nondegradative Pathways

The identification of structures allowing membrane tethering of two organelles into close proximity has instigated considerable research interest into the (patho)physiologic role of inter-organelle contact sites (reviewed in [79]). Indeed, the juxtaposition (<30nm) of membranes of identical (homotypic contacts) or distinct organelles/membrane types (heterotypic contacts) acts as a domain for inter-organelle communication [79].

Membrane adhesion at contact sites is enacted by different protein classes (e.g., tethering, functional, regulatory proteins) [80]. These inter-organelle contacts enable metabolite shuttling, regulation of mitochondrial dynamics, and several cellular housekeeping processes [81,82]. The tethering of contact sites between mitochondria and lysosomes is regulated by multiple proteins under the control of RAB7 [14]. As discussed earlier, RAB7 modulates the tethering and untethering of mitochondrial-lysosomal contact sites through its ability of shifting between an active, lysosomal-localized GTP-binding state and an inactive, cytosolic GDP-binding state.

Lysosomes establish contacts with mitochondria and remain stably tethered for an average of 60 s [14]. Mitochondrial-lysosomal contacts allow the bidirectional regulation of mitochondrial and lysosomal dynamics [14]. These structures do not involve metabolite transfer between tethering organelles nor are they required for autophagosome biogenesis or mitophagy as shown by the fact that they are void of autophagosomal markers (e.g., unc-51 like autophagy activating kinase (ULK1), Atg5, Atg12, and LC3) [14]. The independence of mitochondrial-lysosomal contact sites from mitophagy has been further confirmed in cells knocked out for autophagy receptors (i.e., NDP52, OPTN, neighbor of BRCA1 gene 1 protein (NBR1), tax1-binding protein 1 (TAX1BP1), and p62) in which the genetic ablation does not interfere with mitochondrial-lysosomal contact formation [83].

Recently, mitochondrial-lysosomal contact sites have shown to regulate the rate of mitochondrial fission [84]. Indeed, most fission events are marked by lysosomal-associated membrane protein 1 (LAMP-1)-positive vesicles, but not early endosomes or peroxisomes [14]. A novel murine isoform of DRP1 containing four alternative exons, Drp1_ABCD_, has been identified [84]. DRP1_ABCD_ is located in late endosomes, the plasma membrane, and in association with LAMP-1-positive vesicles at the interface between mitochondria and lysosomes [84]. DRP1_ABCD_ localization relies upon acidification of late endosomes and lysosomes, which suggests additional roles for DRP1 isoforms at mitochondrial-lysosomal contact sites [84]. In yeast, the equivalent of the mammalian mitochondrial-lysosomal contact site, termed vacuole and mitochondria patch (vCLAMP), contains several tethering proteins, including translocase of outer mitochondrial membrane 40 (TOM40), mitochondrial distribution and morphology 10 (MDM10) complementing protein 1 (MCP1), and vacuole sorting protein (VPS) 13 and 39 [85]. Whether the mammalian orthologs of such proteins are involved in establishing mitochondrial-lysosomal contacts is yet to be determined.

The best studied heterotypic contacts are those involving the ER, including ER-mitochondrial, ER-Golgi, ER-peroxisomes, and ER-lipid droplets (LDs) contacts. However, several other contacts not involving ER have been reported, such as LDs-peroxisomes, mitochondria-vacuoles/endosomes/lysosomes, mitochondria-LDs, mitochondria-peroxisomes, and mitochondrial inner and outer membranes (reviewed in [80]).

High-electron microscopy and super-resolution optical microscopy identified ER-mitochondrial contact sites as parallel juxtaposition between mitochondria and smooth or rough ER tubules at a distance ranging from 10 to 80 nm [86,87,88,89,90,91]. Length, thickness, and protein composition of contact zones vary according to the signaling pathway for which contact sites are established (e.g., apoptosis, ER stress response, metabolic dysfunction) [92]. Protein constituents of ER-mitochondrial contact sites include MFN2, phosphoacidic cluster sorting protein 2 (PACS2), vesicle-associated membrane protein-associated protein B (VAPB), protein tyrosine phosphatase interacting protein 51 (PTPIP51), 1,4,5-triphosphate receptor subtype 3 (IP3R3), and voltage-dependent anion channel (VDAC) [93,94,95,96,97,98,99].

The number of ER-mitochondrial contacts depends on the expression of the IP3R Ca^2+^ channel [100]. This channel forms a tether with VDAC via the mitochondrial chaperone glucose-regulated protein 75 (GRP75). The resulting channel modulates Ca^2+^ flux between the ER and the mitochondrial intermembrane space [99]. The ER B-cell-receptor-associated protein 31 (BAP31) is another contact point that interacts with FIS1. The BAP31-FIS1 complex bridges the ER-mitochondrial interface and regulates the induction of apoptosis [101]. Through tethering with the ER, mitochondria also acquire lipid precursors, such as phosphatidylserine and phosphatidic acid, that are subsequently harnessed to synthesize membrane phospholipids, including phosphatidylethanolamine, phosphatidylglycerol, and cardiolipin [102].

The number of ER-mitochondrial contact sites was found to be lower in senescent cells [103]. This observation led to hypothesize that disruption of these contacts may have important implications for cell function and longevity [104]. In asymmetrically dividing cells, noxious protein aggregates are unevenly segregated, such that they are preferentially retained within the mother cell [105]. Here, protein aggregates localize at the ER surface and at ER-mitochondrial contact sites for subsequent disposal within mitochondria [105,106]. In the setting of mitochondrial dysfunction, the clearance of protein aggregates and misfolded proteins becomes impaired, thus favoring their accumulation at the ER-mitochondrial interface [104]. Interestingly, toxic proteins associated with age-related neurological and metabolic disorders, such as α-synuclein, Parkin and protein deglycase, have been located at ER-mitochondrial contact sites [105,107,108]. This abnormal protein distribution interferes with mitochondrial Ca^2+^ uptake from the ER and the execution of autophagy [109]. If protracted, this process may impair cellular bioenergetics and increase reactive oxygen species (ROS) generation by mitochondria and the ER, which further depresses quality control systems [109]. The relevance of oxidative stress as a mechanism in aging and age-related conditions is illustrated in Section 3.

Homotypic contact sites have been described in living cells under real-time imaging experiments showing formation of a peroxisomal reticulum [110]. However, such structures represent organelle fusion intermediates with features different from those found in the heterotypic contacts described above and will not be discussed here.

## 3. Endoplasmic Reticulum-Mitochondrial Contact Sites, Oxidative Stress, and Mitochondrial Quality Control

Dysfunction of the mitochondrial electron transport chain (ETC) and excessive ROS generation have been indicated as primary contributors to aging [3]. ROS are mainly generated as a bioproduct of mitochondrial respiration. Under physiologic conditions, mitochondrial-derived ROS function as intracellular signaling molecules that stimulate defense mechanisms by inducing an adaptive response, a process called mitohormesis [111]. Mitohormesis operates via leakage of hydrogen peroxide as a warning system which sends retrograde signal to nuclear-targeted cytosolic pathways [25]. Under oxidative stress conditions, instead, loss of ROS signal localization and disruption of cell homeostasis occur [112].

The installment of oxidative stress is deleterious for the cell as it induces damage to its constituents, especially mitochondria. Indeed, as a major source of ROS, mitochondria are an immediate target of oxidative damage. In particular, oxidative stress can induce mtDNA base modifications, abasic sites, single- and double-strand breaks, point mutations, large-sized deletions, and changes in mtDNA content [113,114]. While it is well-known that mitochondria produce ROS at the level of complexes I and III [115], ER is also a relevant oxidant source mainly produced by members of cytochrome P450 and nicotinamide adenine dinucleotide phosphate reduced form (NADPH) oxidase 4 (NOX4) [116,117].

Similar to the mitochondrial-lysosomal axis, contacts between mitochondria and the ER can mediate bidirectional signaling to determine the cell’s fate during aging. The transmembrane protein kinase RNA-like ER kinase (PERK), a member of the ER stress/unfolded protein response (UPR) machinery [117], enables ER-mitochondrial tethering during ER stress to facilitate Ca^2+^ influx and ROS-dependent mitochondrial-mediated apoptosis [118]. Moreover, a strongly oxidizing environment around ER-mitochondrial contacts modulates organelle apposition through the mitogen–activated protein kinase (MAPK)-dependent control of mitochondrial mobility [119]. Additional proteins can localize at the level of mitochondria following oxidative stress insults [120]. One of such proteins is the 66 kDa Src homologous-collagen homolog (SHC) isoform, a negative regulator of the epidermal growth factor (EGF)-stimulated MAPK pathway that controls oxidative stress and lifespan in mammals [121].

Mitigation of oxidative stress is achieved via MQC processes. Notably, members of the MQC machinery, in particular those involved in mitochondrial dynamics and autophagy, have been localized at ER-mitochondrial contact sites [24,122]. Here, DRP1 and ER-localized inverted formin 2 (INF2) allow execution of mitochondrial fission by generating a constriction ring around the organelle [123]. On the other hand, the localization of MFN1 and MFN2 fusion proteins at ER-mitochondrial contact sites stabilizes the contact structure [119]. Hence, ER-mitochondrial contact sites may support MQC processes for the removal of dysfunctional mitochondria [123].

Fusion processes mediated by MFN2 are also crucial for the initiation of mitophagy by favoring PINK/Parkin interaction [124,125]. The genetic ablation of MFN2 in fibroblasts, cardiomyocytes, and neurons induces impairment of mitophagy, accumulation of dysfunctional mitochondria, and cell death [126]. Notably, molecular mechanisms involving MFN2-related mitophagy dysfunction have shown to be in place in the setting of several age-related diseases (i.e., Alzheimer’s disease, PD, diabetes, and cardiovascular disease) [126]. Declines in MFN2 protein expression, engulfment of the mitophagic pathway, and accumulation of dysfunctional mitochondria have also been reported in skeletal muscles of older adults with physical frailty and sarcopenia [127]. In further support to the relevance of fusion to the preservation of mitochondrial function during aging, a shift of mitochondrial dynamics signaling toward fusion in muscles of very old hip-fractured patients has been described [128]. In keeping with this view are also findings obtained in mtDNA-mutator mice, a rodent model of premature aging obtained by expressing a proofreading-deficient version of the mtDNA polymerase gamma (PolG mice) [129]. The sarcopenic phenotype of prematurely aged PolG mice is characterized by higher FIS1 expression and increased mitophagy [129].

## 4. Mitochondrial Quality Control Failure as a Mechanism in Inflammaging

Competent MQC pathways, including those involving nondegradative mitochondrial-lysosomal tethering, are instrumental to cell and organismal homeostasis. This is reflected by the tight relationship among mitochondrial dysfunction, redox imbalance, and inflammation during aging [130]. Indeed, under specific circumstances, redox-sensitive inflammatory pathways involving mitochondrial Ca^2+^ metabolism, iron handling, and ROS production may be triggered [131,132]. This impairs the function of the ETC, thereby enhancing oxidant generation. The resulting ROS burst acts as a major pro-inflammatory stimulus through activation of nuclear factor κB (NF-κB) and downstream inflammatory response [133].

Different outcomes ensue depending on the severity of inflammation and the efficiency of cellular quality control systems. In response to moderate inflammatory stimuli and overwhelmed cellular repair systems, an apoptotic cascade may be triggered [134]. On the other hand, the installment of severe inflammation, mitochondrial dysfunction, and ROS-induced damage may culminate in necrosis. As a result, cellular constituents, including whole and fragmented mitochondria as either cell-free components or within MDVs, are released into the circulation. Here, DAMPs, in particular mtDNA and damaged mitochondrial components, trigger inflammation by interacting with TLR, NLRP, and cGAS-STING systems [135,136].

The TLR pathway is elicited by the binding of DAMPs to neutrophils, followed by their activation and organization of an inflammatory response via NF-κB signaling [137]. An alternative mtDNA-induced inflammatory pathway operates through inflammasomes, in particular via the NLRP3 inflammasome [138,139]. NLRP3 has been implicated in a wide range of diseases, including Alzheimer’s disease, cardiovascular disease, asthma, inflammatory bowel disease, nonalcoholic fatty liver disease and nonalcoholic steatohepatitis, graft-versus-host disease, type 1 diabetes, rheumatoid arthritis, myelodysplastic syndrome, gout, etc. (reviewed in [140]). NLRP3 consists of a group of cytosolic protein complexes, the activation of which results in the engagement of caspase-1 [141]. The latter subsequently cleaves and activates interleukin (IL) 1 and 18. It is noteworthy that redox-sensitive inflammatory and inflammasome-mediated pathways may act synergistically to reinforce inflammation [142].

Albeit the exact mechanisms linking inflammasome activation to inflammaging remain elusive, bacterial-like motifs within the mtDNA that are sensed by NLRs are major suspects [143]. What is more, NLRP3 activators can trigger a self-maintaining circle by inducing mitochondrial dysfunction, ROS bursts, and consequent mtDNA damage [139]. Upon release, oxidized mtDNA may function as the ultimate NLRP3 ligand [139]. Hence, inflammasomes, including NLRP3, may represent key up-stream checkpoints of the innate immune system during the development of inflammaging.

The cGAS-STING DNA-sensing pathway is an additional component of the innate immune system [136]. Upon binding to mtDNA, cGAS proceeds through STING protein recruitment which triggers the phosphorylation of the transcription factor interferon (IFN) regulatory factor 3 (IRF-3) via TANK-binding kinase (TBK). Activated IRF-3 induces the production of type I and III IFN and IFN-stimulated nuclear gene products. A persistent activation of the cGAS-STING pathway has been invoked as a mechanism in inflammaging by promoting cellular senescence through IFN-mediated induction of p53 [144,145,146]. Upon activation of the senescence program, cell cycle arrest ensues. Though, the cell remains metabolically active and undergoes functional changes characterized by a peculiar protein expression and secretion phenotype, known as senescence-associated secretory phenotype (SASP) [147]. The SASP fingerprint includes ILs, chemokines, growth factors, secreted proteases, and secreted extracellular matrix components [147]. SASP factors modify the local microenvironment through autocrine and paracrine actions to ensure prevention of growth of damaged cells, recruitment of immune cells, and promotion of tissue repair [148,149]. On the other hand, the insufficient clearance of senescent cells during aging may feed systemic inflammation through sustained production of SASP-related pro-inflammatory cytokines, including IL1β, IL6, and IL8 [150].

## 5. Circulating Mitochondrial-Derived Vesicles, Systemic Inflammation, and Neurodegeneration: The Case of Parkinson’s Disease

PD has a multifaceted pathophysiology that recapitulates all major hallmarks of aging and has therefore been proposed as a prototypical geroscience condition [4,129,151]. In this complex scenario, mitochondrial dysfunction in neurons and systemic inflammation are invoked as major pathogenic mechanisms in PD [152,153]. Although the molecular events linking these two processes are yet to be disentangled, failing MQC and the release of mitochondrial DAMPs within small EVs (sEVs) have recently been associated with a specific inflammatory profile in older adults with PD [71]. In particular, older people with PD showed greater serum concentrations of mixed sEVs compared with non-PD peers [71]. The characterization of sEVs revealed their identity as exosomes of endosomal origin deriving from the fusion of multivesicular bodies (MVBs) with the plasma membrane [71]. Notably, mitochondrial signatures, including adenosine triphosphate 5A (ATP5A), nicotinamide adenine dinucleotide reduced form (NADH):ubiquinone oxidoreductase subunit S3 (NDUFS3), and succinate dehydrogenase complex iron sulfur subunit B (SDHB), were identified in purified sEVs from older adults with PD. This finding indicates the presence of MDVs among sEVs in PD [71]. More relevant was the observation that levels of MDVs were lower in older people with PD relative to non-PD controls [71]. The generation and release of MDVs are orchestrated by mitochondrial-lysosomal crosstalk and may be triggered as a mechanism to dispose dysfunctional organelles, the persistence of which would be detrimental for cell homeostasis [16]. According to this view, the increased sEV secretion detected in PD might reflect the cell’s attempt to clear out dysfunctional mitochondria. The lower secretion of MDVs may therefore be indicative of MQC stalling in this condition.

EV cargoes enriched in damaged mitochondria may also be delivered to lysosomes for degradation [66]. In support to this hypothesis, alterations of lysosomal function were described in association with impaired mitochondrial biogenesis in fibroblasts from a young PD patient with Parkin gene (*PARK2*) mutation [154].

Relevant insights into the association of mitochondrial dysfunction with systemic inflammation in PD were provided by the integrated analysis of mitochondrial and inflammatory markers. This approach revealed a molecular fingerprint of PD, encompassing MDV markers and inflammatory biomolecules [71]. The presence of fibroblast growth factor 21 (FGF21) within the biomarker profile of PD is especially noticeable. Indeed, FGF21 has recently been related to dysfunctional MQC in neurons and has shown to be induced in brains of murine models of tauopathy and prion disease [155]. Thus, it is proposed that FGF21 may function as a “mitokine” and serve as a biomarker of mitochondrial dysfunction in the brain [155].

The liaison among failing mitochondrial fidelity pathways, MDV secretion, and systemic inflammation may not be exclusively involved in neurodegeneration. Indeed, other conditions such as HIV infection are characterized by pyroptotic bystander cell death and release of DAMPs that may trigger the same pathways as those identified in PD and inflammaging [156]. In addition, a massive release of DAMPs is acknowledged as a factor in the development of multiorgan failure in patients with severe injuries or during hemorrhagic shock [157]. Mitochondrial DNA scavenging through the injection of hexadimethrine bromide has shown to prevent the mtDNA surge in the circulation and to rescue multiorgan failure in a preclinical model of tissue injury and hemorrhagic shock [157]. Although the pathophysiology of the multiple organ failure syndrome is different from that of inflammaging and neurodegeneration, the release of mitochondrial DAMPs may be a converging mechanism underpinning all these conditions. The scavenging of circulating mitochondrial DAMPs, including mtDNA, might therefore represent a yet unexplored therapeutic option for the management of age-related conditions.

## 6. Conclusions

Mitochondrial dysfunction, arising from failure of mitochondrial fidelity pathways, is a major mechanism driving aging and the development of age-related diseases. In this context, MQC processes may represent ideal targets for geroprotective interventions. Notably, many of the proteins involved in MQC pathways have been localized at inter-organelle interface. Such contact sites may therefore participate to some of the processes responsible for cell dyshomeostasis triggered by mitochondrial dysfunction. Hence, a deeper characterization of the structures ensuring inter-organelle crosstalk is crucial for a comprehensive assessment of mitochondrial dysfunction during aging [158]. This knowledge, in turn, is necessary to unveil strategic pathways that may be targeted for geroprotective interventions.
